# Polymorphisms of matrix metalloproteinases affect the susceptibility of esophageal cancer: Evidence from 20412 subjects, systematic review and updated meta-analysis

**DOI:** 10.1097/MD.0000000000027229

**Published:** 2021-09-24

**Authors:** Hai Chen, Xianquan Xu, Congshu Hua, Heng Zhang, Junling Jian, Tengfei Ge, Jianfeng Xie, Zaicheng Yu

**Affiliations:** a Department of Cardiothoracic Surgery, Anhui Chest Hospital, Anhui, China; b The First Department of Thoracic Surgery, Anhui Chest Hospital, Anhui, China; c The Third Department of Thoracic Surgery, Anhui Chest Hospital, Anhui, China; d Department of Thoracic Surgery, the First Affiliated Hospital of Anhui Medical University, Anhui, China.

**Keywords:** cancer risk, esophageal cancer, matrix metalloproteinases, polymorphisms

## Abstract

**Background:**

The results of how matrix metalloproteinases (MMPs) polymorphisms affect esophageal cancer (EC) risk are not consistent, especially for MMP1,2,7 and 9. A meta-analysis focused on the impact of MMPs to digestive cancers, but not a precise analysis to EC, therefore, we designed the current study to make a clear understanding of the association between MMPs polymorphisms and EC.

**Methods:**

Up to March 2020, we searched several databases to find case-control cohorts concerned about the risk of MMPs polymorphisms to EC risk. Odds ratios with 95% confidence intervals under five genetic models to generate the risk predicted value. The *Q* test and *I*^2^ statistics are used to estimate heterogeneity. Sensitivity analysis, Egger test, and Begg's funnel plot were employed to assess the results. In-silico analysis was performed to study the association between the polymorphism and mRNA expression.

**Results:**

19 case-control studies were enrolled, including 8371 EC patients and 12041 health controls. We observed the increased risk in BA vs. AA and BB + BA vs. AA models of MMP1-rs1799750 polymorphism. The protective effectiveness of EC was found in the MMP2 rs243865 polymorphism in B vs. A, BA vs. AA, and BB + BA vs. AA models. Meanwhile, the risk effect was also observed in the MMP7 rs11568818 polymorphism in most genetic models. In the furthermore bioinformatics analysis, we found that MMP1, MMP3, MMP7, MMP9, MMP12, MMP13 all increased in the tumor tissues, and the genetic alteration in the polymorphisms could impact the mRNA expression of the above MMPs.

**Conclusion:**

MMP1 rs1799705 and MMP7 rs1156818 polymorphisms will take part in the tumorigenesis of EC, while MMP2 rs243865 acts as a protective role to decrease the risk of EC.

## Abstract 2

Supplemental Digital Content is available in the text

## 1 Introduction

Esophageal cancer (EC), consisting of two major histological types, esophageal squamous cell carcinoma (ESCC) and esophageal adenocarcinoma (EAC), is the seventh most frequent cancer globally, and causes the sixth cancer-specific death, based on the data from World Health Organization, and Eastern Asia accounts for the highest rate of both incidence and mortality.^[1]^ The ESCC type of EC is more common in China and accounts for more than 90% new cases, while the EAC type is more common in western countries.^[2,3]^ The newly diagnosed ESCC patients are mostly living in China around Taihang mountain and Nanao island, the annual average age-standardized incidence of ESCC is high to 208/100,000.^[4,5]^ As to the United States, there are about 17,650 new cases and 16,080 estimated deaths in 2019 and leads to a heavy healthy burden.^[6]^ Several researchers reported that EC development is dependent upon several factors, such as genetic susceptibility, environment, alcohol consumption, cigarette smoking.^[5,7]^

Hou et al^[8]^ reported that EC patients from hospital-based studies showed a higher survival rate while comparing with the population-based surveillance, Chen et al^[9]^ also drew out that individuals aged 40 to 69 years in high risk areas of EC showed a significant decrease incidence and mortality. Therefore, it is necessary to find the biomarkers to distinguish the different susceptibility of EC. Zhang et al^[10]^ reported that Matrix metalloproteinase 2 (MMP2) -1306 (rs243865) had a decreased incidence of ESCC, this result was also confirmed by Yu et al^[11]^ 's study. However, Haniehsadat et al^[12]^ revealed that No statistically significant differences were observed in the genotype and allele frequencies of MMP2 (-1306C/T) and MMP9 (-1562C/T) between patients and controls. Li et al^[13]^ made a meta-analysis among the digestive cancers in 2013, and they reported that MMP1 and MMP7 are the risk factors for digestive cancers, while MMP2 and MMP9 play protect roles, however the precision role of EC patients were not conducted. Due to this kind of consistent, we designed a study to enroll all the publications mentioning the matrix metalloproteinases (MMPs) polymorphisms and susceptibility of EC, to try to generate a more convincing conclusion. And we also used bioinformatics analysis to find the potential reason for the effect of MMPs polymorphisms on EC.

## 2 Materials and methods

### 2.1 Cohorts searching

Up to March 2020, two investigators of our team searched through the Web of Science, Google Scholar, Wanfang database, PubMed, Embase, and CNKI databases to find the case-control cohorts which meet our criteria. The following search strategy is used for the searching of relevant citations: (matrix metalloproteinases OR MMP) AND (esophageal cancer OR esophageal squamous cell carcinoma OR esophageal adenocarcinoma OR EC OR ESCC OR EA) AND (SNP OR variant OR mutation OR polymorphism OR genotype). This study is a meta-analysis and does not require institutional review board approval or patient consent.

### 2.2 Selection criteria

Articles included in our research must meet the following conditions: (1) study the relationship between EC and MMPs; (2) provide sufficient data of each patient for extraction and calculation; (3) subjects are human patients; (4) the case-control study included control group and EC patients case group. In contrast, studies would be removed if they were: (1) lack of genotype frequency data; (2) case only study, review and meta-analysis, repetitive publications; (3) animal or cell line based studies. When duplicate data appear in different articles, our meta-analysis only includes the latest data for calculation.

### 2.3 Data extraction and quality assessment

These following items are extracted from all the enrolled studies, including disease type, first author, ethnicity, source of control, genotype distribution in cases, and controls, publication year. Any differences were resolved through group discussions until all consensus was reached. The Newcastle–Ottawa Scale (NOS) was applied to assess the quality of all the enrolled studies (http://www.ohri.ca/programs/clinical_epidemiology/oxford.asp).

### 2.4 In-silico analysis

The mRNA expression of MMPs among normal esophagus tissues and EC tissues were compared with the GEPIA (http://gepia.cancer-pku.cn/), which contains the data of gene expression from the TCGA and the GTEx projects.^[14]^

### 2.5 Statistical analysis

The meta-statistical analysis were performed using STATA 12.0. The relationship between EC and MMPs was assessed using pooled odds ratios with 95% confidence intervals under B vs. A, BB vs. AA, BA vs. AA, BB + BA vs. AA, and BB vs. BA + AA models (AA, homozygotes for the major allele; BA, heterozygotes; BB, homozygous for the minor allele). Additionally, we performed a stratified analysis based on disease type, race, source of control, and quality score. The Q test and *I*^*2*^ statistics are used to estimate heterogeneity. The random-effects model is applicable when existing heterogeneity (*P* < .1); otherwise, the fixed effect model is used when *P* > .1. Chi-square test was employed to calculate the Hardy–Weinberg equilibrium of the control group to compare the expected and actual genotype frequencies.^[15]^ Furthermore, the method of evaluating the stability of the overall analysis is sensitivity analysis, and the method of evaluating publication bias is through the Egger test and the Begg's funnel plot.^[16]^ A statistically significant result is a *P* value of less than .05 after adjusting by Bonferroni correction.

## 3 Results

### 3.1 Study characteristics

**Figure 1:**
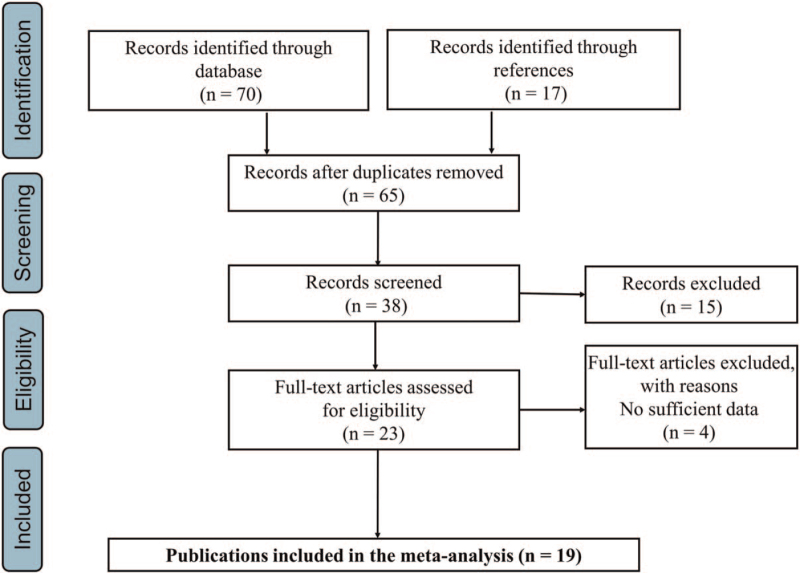
Flowchart of enrolled studies selection procedure.

The search and enrolled process are shown in Figure 1. We firstly grasped 70 studies from the databases, and the other 17 articles were also recorded for consideration after checking the references. Meanwhile, several studies were excluded because of a lack of genotype frequencies or duplicated data. Finally, 19 case-control studies with the genotype frequencies of MMPs were enrolled.^[10–12,17–29]^ The details of demographic characters and genotype frequencies are shown in Table 1. For all the enrolled 19 studies, we conducted the quality evaluation by the NOS scale, all the studies are with high quality for the subsequent analysis (see Supplementary Digital Content Table S1, http://links.lww.com/MD2/A462 which illustrates the quality of the enrolled studies).

**Table 1:** Characteristics of the enrolled studies on MMPs polymorphism and EC.

										Cases			Controls		
MMPs	SNP	First Author	Year	Ethnicity	Genotyping Method	Source of Control	Type	Function to tumorigenesis	HWE	PAA	PAB	PBB	HAA	HAB	HBB
MMP1	rs1799750	Jin et al.	2005	Asians	PCR-RFLP	HB	ESCC	None	N	28	76	130	51	105	194
MMP1	rs1799750	Bradbury et al.	2009	Caucasian	Taqman	PB	EA	Risky	Y	78	153	82	152	210	92
MMP1	rs1799750	Cheung et al.	2012	Caucasian	Taqman	PB	EA	Risky	Y	77	148	84	95	134	50
MMP1	rs1799750	Guan et al.	2014	Asians	PCR	PB	ESCC	None	Y	10	41	81	5	40	87
MMP2	rs243865	Yu et al.	2004	Asians	PCR-RFLP	PB	ESCC	Protective	Y	409	112	6	539	220	18
MMP2	rs243865	Chen et al.	2009	Asians	PCR-RFLP	PB	ESCC	Protective	Y	149	36	3	245	73	6
MMP2	rs243865	Sun et al.	2009	Asians	PCR-RFLP	PB	ESCC	Risky	Y	284	48	3	487	137	6
MMP2	rs243865	Eftekhary et al.	2015	Asians	PCR-RFLP	PB	ESCC	None	N	51	17	1	46	10	4
MMP2	rs243865	Zhang et al.	2015	Asians	PCR-RFLP	HB	ESCC	Protective	Y	136	73	17	136	73	17
MMP2	rs2285053	Yu et al.	2004	Asians	PCR-RFLP	PB	ESCC	Protective	Y	323	179	25	425	313	39
MMP2	rs2285053	Zhang et al.	2007	Asians	PCR-RFLP	PB	ESCC	None	Y	209	94	13	397	186	26
MMP2	rs2285053	Chen et al.	2009	Asians	PCR-RFLP	PB	ESCC	Risky	Y	94	80	14	196	119	9
MMP2	rs2285053	Sun et al.	2009	Asians	PCR-RFLP	PB	ESCC	None	Y	222	100	13	408	187	29
MMP3	rs3025058	Zhang et al.	2004	Asians	PCR-RFLP	PB	ESCC	None	Y	1	73	160	8	105	237
MMP3	rs3025058	Bradbury et al.	2009	Caucasian	Taqman	PB	EA	Risky	Y	60	169	84	118	232	105
MMP3	rs3025058	Cheung et al.	2012	Caucasian	Taqman	PB	EA	None	N	83	167	59	56	159	64
MMP3	rs3025058	Guan et al.	2014	Asians	PCR	PB	ESCC	Protective	Y	20	72	40	6	61	65
MMP3	rs3025058	Zhang et al.	2015	Asians	PCR-RFLP	HB	ESCC	None	N	163	37	26	168	35	23
MMP7	rs11568818	Zhang et al.	2005	Asians	PCR-RFLP	PB	ESCC	None	Y	216	41	1	316	33	1
MMP7	rs11568818	Malik et al.	2011	Asians	PCR-RFLP	PB	ESCC	Risky	Y	36	51	48	63	92	40
MMP9	rs3918242	Fu et al.	2009	Asians	PCR	PB	ESCC	None	Y	174	57	4	239	60	1
MMP9	rs3918242	Guan et al.	2014	Asians	PCR	PB	ESCC	None	Y	84	38	10	92	34	6
MMP9	rs3918242	Zhang et al.	2015	Asians	PCR-RFLP	HB	ESCC	Risky	Y	18	44	164	7	46	173
MMP9	rs3918242	Eftekhary et al.	2015	Asians	PCR-RFLP	PB	ESCC	None	Y	40	25	3	34	23	3
MMP9	rs2250889	Wu et al.	2008	Asians	PCR	PB	ESCC	Risky	N	20	70	42	7	65	60
MMP9	rs2250889	Fu et al.	2009	Asians	PCR	PB	ESCC	Protective	Y	35	39	161	37	120	143
MMP9	rs17576	Wu et al.	2008	Asians	PCR	PB	ESCC	None	Y	11	60	67	7	59	66
MMP9	rs17576	Fu et al.	2009	Asians	PCR	PB	ESCC	None	Y	60	79	96	78	135	87
MMP12	rs2276109	Zhang et al.	2007	Asians	PCR-RFLP	PB	ESCC	None	Y	304	12	0	573	36	0
MMP12	rs2276109	Bradbury et al.	2009	Caucasian	Taqman	PB	EA	Riskey	Y	240	68	5	373	77	5
MMP12	rs2276109	Li et al.	2010	Asians	PCR	PB	ESCC	None	Y	322	13	0	588	36	0
MMP12	rs2276109	Cheung et al.	2012	Caucasian	Taqman	PB	EA	None	Y	238	65	6	226	50	3
MMP12	rs652438	Bradbury et al.	2009	Caucasian	Taqman	PB	EA	None	N	274	38	31	404	46	5
MMP12	rs652438	Cheung et al.	2012	Caucasian	Taqman	PB	EA	None	N	272	34	3	248	28	3
MMP13	rs2252070	Zhang et al.	2007	Asians	PCR-RFLP	PB	ESCC	None	Y	82	164	70	160	316	133
MMP13	rs2252070	Li et al.	2010	Asians	PCR	PB	ESCC	None	Y	89	170	76	163	324	137

EA = esophageal adenocarcinoma, EC = esophageal cancer, ESCC = esophageal squamous cell carcinoma, H-B = hospital-based, MMPs = matrix metalloproteinases, P-B = population-based. *P* > .05 means conformed to HWE; N, polymorphisms did not conform to HWE in the control group;Y, polymorphisms conform to HWE in the control group.

### 3.2 Meta-analysis results

#### 3.2.1 Association between MMP1 rs1799750 polymorphism and EC susceptibility

**Figure 2:**
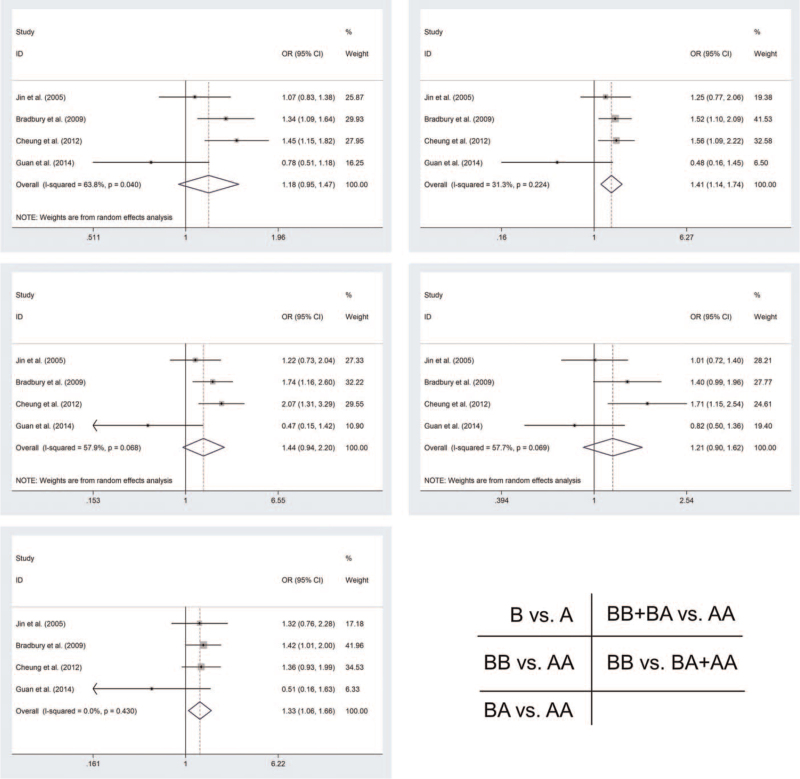
Forest plot showing the risk of MMP1-rs1799750 polymorphism to EC risk.

Table 2 and Figure 2 show the results of pool analysis of MMPs polymorphism and EC sensitivity. According to our calculations, MMP1 rs1799750 polymorphism was associated with sensitivity to EC in two models among the overall analysis (BA vs. AA [OR = 1.325, 95%Cl = 1.057–1.661, P = 0.015]; BB + BA vs. AA [OR = 1.411, 95%Cl = 1.143–1.741, *P* = .001]) and subgroup analysis of PB (BA vs. AA [OR = 1.327, 95%Cl = 1.035–1.7, *P* = .025]; BB + BA vs. AA [OR = 1.449, 95%Cl = 1.148–1.827, *P* = .002]). In addition, we found an important association between MMP1 rs1799750 polymorphism and EAC in the subgroup analysis of Caucasian. (B vs. A [OR = 1.386, 95%Cl = 1.189–1.615, *P* < .001]; BB vs. AA [OR = 1.876, 95% Cl = 1.385–2.542, *P* < .001]; BA vs. AA [OR = 1.394, 95%Cl = 1.08–1.799, *P* = .011]; BB + BA vs. AA [OR = 1.534, 95% Cl = 1.207–1.948, *P* < .001]; BB vs. BA + AA [OR = 1.525, 95%Cl = 1.179–1.972, *P* = .001]).

**Table 2:** Meta-analysis showing the risk of MMPs polymorphisms to EC.

Comparison	Subgroup	N	PH	PZ	Random	Fixed
MMP1-rs1799750						
B vs. A	Overall	4	0.04	0.134	1.182 (0.95–1.471)	1.237 (1.091–1.403)
BB vs. AA	Overall	4	0.068	0.09	1.44 (0.944–2.195)	1.556 (1.209–2.003)
BA vs. AA	Overall	4	0.43	0.015^∗^	1.329 (1.059–1.667)	1.325 (1.057–1.661)
BB + BA vs. AA	Overall	4	0.224	0.001^∗^	1.373 (1.044–1.805)	1.411 (1.143–1.741)
BB vs. BA + AA	Overall	4	0.069	0.213	1.207 (0.898–1.623)	1.222 (1.012–1.474)
B vs. A	Asians/ESCC	2	0.206	0.852	0.953 (0.707–1.286)	0.979 (0.786–1.22)
BB vs. AA	Asians/ESCC	2	0.124	0.920	0.861 (0.347–2.135)	1.024 (0.648–1.616)
BA vs. AA	Asians/ESCC	2	0.148	0.698	0.949 (0.393–2.293)	1.101 (0.677–1.792)
BB + BA vs. AA	Asians/ESCC	2	0.119	0.795	0.885 (0.358–2.19)	1.06 (0.681–1.65)
BB vs. BA + AA	Asians/ESCC	2	0.512	0.690	0.945 (0.716–1.247)	0.945 (0.716–1.247)
B vs. A	Caucasian/EAC	2	0.614	<0.001^∗^	1.386 (1.189–1.615)	1.386 (1.189–1.615)
BB vs. AA	Caucasian/EAC	2	0.572	<0.001^∗^	1.875 (1.384–2.541)	1.876 (1.385–2.542)
BA vs. AA	Caucasian/EAC	2	0.875	0.011^∗^	1.394 (1.08–1.799)	1.394 (1.08–1.799)
BB + BA vs. AA	Caucasian/EAC	2	0.917	<0.001^∗^	1.534 (1.208–1.948)	1.534 (1.207–1.948)
BB vs. BA + AA	Caucasian/EAC	2	0.447	0.001^∗^	1.522 (1.176–1.97)	1.525 (1.179–1.972)
B vs. A	PB/Y	3	0.036	0.183	1.212 (0.913–1.61)	1.294 (1.121–1.494)
BB vs. AA	PB/Y	3	0.052	0.181	1.477 (0.834–2.616)	1.685 (1.261–2.252)
BA vs. AA	PB/Y	3	0.252	0.025^∗^	1.297 (0.944–1.784)	1.327 (1.035–1.7)
BB + BA vs. AA	PB/Y	3	0.130	0.002^∗^	1.362 (0.928–1.998)	1.449 (1.148–1.827)
BB vs. BA + AA	PB/Y	3	0.076	0.185	1.291 (0.885–1.885)	1.339 (1.066–1.682)
MMP2-rs243865						
B vs. A	Overall	5	0.255	<0.001^∗^	0.773 (0.649–0.922)	0.761 (0.659–0.88)
BB vs. AA	Overall	5	0.55	0.133	0.72 (0.446–1.164)	0.698 (0.437–1.116)
BA vs. AA	Overall	5	0.147	0.001^∗^	0.775 (0.611–0.982)	0.743 (0.628–0.879)
BB + BA vs. AA	Overall	5	0.209	<0.001^∗^	0.756 (0.613–0.933)	0.735 (0.625–0.865)
BB vs. BA + AA	Overall	5	0.567	0.182	0.752 (0.468–1.209)	0.729 (0.458–1.16)
B vs. A	PB	4	0.679	<0.001^∗^	0.707 (0.599–0.834)	0.706 (0.598–0.832)
BB vs. AA	PB	4	0.671	0.056	0.549 (0.287–1.05)	0.535 (0.281–1.016)
BA vs. AA	PB	4	0.233	<0.001^∗^	0.717 (0.563–0.913)	0.698 (0.58–0.841)
BB + BA vs. AA	PB	4	0.429	<0.001^∗^	0.687 (0.573–0.823)	0.686 (0.572–0.822)
BB vs. BA + AA	PB	4	0.626	0.087	0.589 (0.308–1.125)	0.571 (0.301–1.084)
B vs. A	Y	4	0.165	<0.001^∗^	0.768 (0.629–0.938)	0.756 (0.652–0.876)
BB vs. AA	Y	4	0.584	0.235	0.762 (0.466–1.246)	0.746 (0.46–1.21)
BA vs. AA	Y	4	0.253	<0.001^∗^	0.733 (0.596–0.902)	0.722 (0.608–0.857)
BB + BA vs. AA	Y	4	0.204	<0.001^∗^	0.735 (0.594–0.91)	0.721 (0.61–0.851)
BB vs. BA + AA	Y	4	0.668	0.324	0.8 (0.492–1.301)	0.786 (0.487–1.269)
MMP2-rs2285053						
B vs. A	Overall	4	0.009	0.903	1.015 (0.804–1.28)	0.968 (0.863–1.086)
BB vs. AA	Overall	4	0.053	0.658	1.13 (0.658–1.938)	1.042 (0.755–1.437)
BA vs. AA	Overall	4	0.048	0.812	0.971 (0.764–1.235)	0.93 (0.806–1.074)
BB + BA vs. AA	Overall	4	0.016	0.961	0.993 (0.764–1.292)	0.942 (0.821–1.081)
BB vs. BA + AA	Overall	4	0.123	0.659	1.125 (0.709–1.786)	1.074 (0.781–1.477)
MMP3-rs3025058						
B vs. A	Overall	5	<0.001	0.592	0.926 (0.698–1.227)	0.971 (0.862–1.094)
BB vs. AA	Overall	5	<0.001	0.716	0.875 (0.426–1.798)	0.97 (0.748–1.258)
BA vs. AA	Overall	5	0.009	0.87	0.959 (0.583–1.578)	1.013 (0.809–1.27)
BB + BA vs. AA	Overall	5	0.001	0.771	0.923 (0.537–1.584)	1.008 (0.819–1.24)
BB vs. BA + AA	Overall	5	0.017	0.484	0.89 (0.641–1.234)	0.928 (0.774–1.112)
B vs. A	Asians/ESCC	3	0.002	0.555	0.866 (0.538–1.395)	0.891 (0.736–1.08)
BB vs. AA	Asians/ESCC	3	0.001	0.866	0.871 (0.177–4.288)	0.809 (0.512–1.277)
BA vs. AA	Asians/ESCC	3	0.031	0.959	0.971 (0.319–2.953)	0.95 (0.624–1.445)
BB + BA vs. AA	Asians/ESCC	3	0.006	0.901	0.922 (0.256–3.325)	0.939 (0.657–1.342)
BB vs. BA + AA	Asians/ESCC	3	0.016	0.464	0.811 (0.462–1.423)	0.84 (0.649–1.087)
B vs. A	Caucasian/EAC	2	0.007	0.991	1.002 (0.662–1.518)	1.024 (0.88–1.192)
BB vs. AA	Caucasian/EAC	2	0.005	0.996	0.998 (0.402–2.477)	1.058 (0.772–1.451)
BA vs. AA	Caucasian/EAC	2	0.011	0.972	1.012 (0.508–2.018)	1.041 (0.796–1.361)
BB + BA vs. AA	Caucasian/EAC	2	0.004	0.981	1.009 (0.475–2.146)	1.045 (0.81–1.348)
BB vs. BA + AA	Caucasian/EAC	2	0.101	0.862	0.999 (0.654–1.526)	1.023 (0.793–1.32)
B vs. A	Y	3	<0.001	0.683	0.904 (0.556–1.469)	1.022 (0.876–1.192)
BB vs. AA	Y	3	<0.001	0.997	1.003 (0.182–5.545)	1.156 (0.807–1.657)
BA vs. AA	Y	3	0.012	0.845	1.128 (0.339–3.755)	1.248 (0.899–1.732)
BB + BA vs. AA	Y	3	0.001	0.930	1.066 (0.259–4.386)	1.224 (0.897–1.669)
BB vs. BA + AA	Y	3	0.004	0.556	0.854 (0.504–1.446)	0.945 (0.761–1.174)
B vs. A	N	2	0.111	0.264	0.931 (0.676–1.281)	0.898 (0.744–1.085)
BB vs. AA	N	2	0.113	0.246	0.83 (0.45–1.533)	0.8 (0.548–1.166)
BA vs. AA	N	2	0.194	0.263	0.853 (0.562–1.295)	0.835 (0.61–1.144)
BB + BA vs. AA	N	2	0.089	0.57	0.869 (0.536–1.409)	0.858 (0.647–1.137)
BB vs. BA + AA	N	2	0.311	0.486	0.891 (0.636–1.247)	0.889 (0.639–1.237)
MMP7-rs11568818						
B vs. A	Overall	2	0.644	0.001^∗^	1.578 (1.219–2.044)	1.578 (1.219–2.044)
BB vs. AA	Overall	2	0.803	0.013^∗^	2.068 (1.165–3.67)	2.068 (1.166–3.669)
BA vs. AA	Overall	2	0.089	0.351	1.34 (0.725–2.479)	1.362 (0.95–1.952)
BB + BA vs. AA	Overall	2	0.36	0.014^∗^	1.541 (1.094–2.171)	1.538 (1.091–2.168)
BB vs. BA + AA	Overall	2	0.752	0.003^∗^	2.108 (1.295–3.432)	2.108 (1.295–3.431)
MMP9-rs3918242						
B vs. A	Overall	4	0.046	0.761	1.055 (0.746–1.494)	1.049 (0.854–1.29)
BB vs. AA	Overall	4	0.044	0.877	1.091 (0.364–3.27)	0.877 (0.501–1.534)
BA vs. AA	Overall	4	0.118	0.613	0.992 (0.643–1.53)	1.076 (0.81–1.429)
BB + BA vs. AA	Overall	4	0.055	0.977	0.993 (0.619–1.593)	1.106 (0.843–1.451)
BB vs. BA + AA	Overall	4	0.251	0.867	1.107 (0.609–2.012)	0.969 (0.669–1.403)
B vs. A	PB	3	0.49	0.065	1.27 (0.986–1.635)	1.269 (0.986–1.634)
BB vs. AA	PB	3	0.411	0.136	1.766 (0.774–4.033)	1.84 (0.825–4.103)
BA vs. AA	PB	3	0.72	0.22	1.207 (0.894–1.63)	1.207 (0.894–1.629)
BB + BA vs. AA	PB	3	0.608	0.113	1.264 (0.946–1.689)	1.264 (0.946–1.688)
BB vs. BA + AA	PB	3	0.444	0.161	1.695 (0.749–3.836)	1.765 (0.797–3.908)
MMP9-rs2250889						
B vs. A	Overall	2	<0.001	0.966	0.98 (0.377–2.543)	1.108 (0.894–1.372)
BB vs. AA	Overall	2	0.004	0.475	0.569 (0.121–2.674)	0.801 (0.518–1.24)
BA vs. AA	Overall	2	0.868	<0.001^∗^	0.353 (0.215–0.579)	0.354 (0.215–0.582)
BB + BA vs. AA	Overall	2	0.072	0.188	0.542 (0.218–1.349)	0.63 (0.411–0.965)
BB vs. BA + AA	Overall	2	<0.001	0.829	1.17 (0.282–4.845)	1.459 (1.101–1.934)
MMP9-rs17576						
B vs. A	Overall	2	0.124	0.163	1.11 (0.792–1.555)	1.156 (0.943–1.416)
BB vs. AA	Overall	2	0.155	0.27	1.1 (0.526–2.298)	1.255 (0.838–1.878)
BA vs. AA	Overall	2	0.774	0.143	0.742 (0.497–1.107)	0.741 (0.497–1.106)
BB + BA vs. AA	Overall	2	0.392	0.827	0.962 (0.669–1.383)	0.961 (0.669–1.379)
BB vs. BA + AA	Overall	2	0.056	0.38	1.291 (0.73–2.283)	1.366 (1.026–1.82)
MMP12-rs2276109						
B vs. A	Overall	4	0.069	0.923	1.018 (0.712–1.455)	1.115 (0.903–1.377)
BA vs. AA	Overall	4	0.078	0.984	0.996 (0.684–1.451)	1.078 (0.856–1.359)
BB + BA vs. AA	Overall	4	0.066	0.969	1.008 (0.688–1.475)	1.101 (0.878–1.381)
B vs. A	Asians/ESCC	2	0.92	0.067	0.651 (0.411–1.031)	0.651 (0.411–1.031)
BA vs. AA	Asians/ESCC	2	0.919	0.064	0.644 (0.404–1.026)	0.644 (0.404–1.026)
BB + BA vs. AA	Asians/ESCC	2	0.919	0.064	0.644 (0.404–1.026)	0.644 (0.404–1.026)
B vs. A	Caucasian/EAC	2	0.829	0.027^∗^	1.315 (1.032–1.676)	1.314 (1.031–1.675)
BB vs. AA	Caucasian/EAC	2	0.834	0.26	1.699 (0.669–4.314)	1.706 (0.674–4.317)
BA vs. AA	Caucasian/EAC	2	0.705	0.053	1.31 (0.997–1.721)	1.309 (0.997–1.72)
BB + BA vs. AA	Caucasian/EAC	2	0.758	0.034^∗^	1.333 (1.023–1.738)	1.333 (1.022–1.738)
BB vs. BA + AA	Caucasian/EAC	2	0.817	0.309	1.612 (0.636–4.086)	1.618 (0.641–4.087)
B vs. A	Y	4	0.069	0.923	1.018 (0.712–1.455)	1.115 (0.903–1.377)
BA vs. AA	Y	4	0.078	0.984	0.996 (0.684–1.451)	1.078 (0.856–1.359)
BB + BA vs. AA	Y	4	0.066	0.969	1.008 (0.688–1.475)	1.101 (0.878–1.381)
MMP12-rs652438						
B vs. A	Overall	2	0.003	0.238	1.692 (0.706–4.053)	1.914 (1.454–2.52)
BB vs. AA	Overall	2	0.015	0.317	3.171 (0.331–30.386)	5.462 (2.539–11.754)
BA vs. AA	Overall	2	0.789	0.376	1.169 (0.828–1.652)	1.169 (0.827–1.652)
BB + BA vs. AA	Overall	2	0.064	0.175	1.506 (0.833–2.724)	1.587 (1.164–2.163)
BB vs. BA + AA	Overall	2	0.016	0.321	3.121 (0.329–29.584)	5.372 (2.498–11.552)
MMP13-rs2252070						
B vs. A	Overall	2	0.959	0.894	1.009 (0.882–1.154)	1.009 (0.882–1.154)
BB vs. AA	Overall	2	0.969	0.88	1.021 (0.777–1.343)	1.021 (0.777–1.343)
BA vs. AA	Overall	2	0.822	0.902	0.986 (0.785–1.238)	0.986 (0.785–1.238)
BB + BA vs. AA	Overall	2	0.857	0.974	0.996 (0.803–1.236)	0.996 (0.803–1.236)
BB vs. BA + AA	Overall	2	0.918	0.793	1.031 (0.821–1.296)	1.031 (0.821–1.295)

EC = esophageal cancer, MMPs = matrix metalloproteinases, N = polymorphisms did not conform to HWE in the control group, P-B = population-based, PH = P-value of Q test for heterogeneity test, PZ = means statistically significant (*P*<.05), Y = polymorphisms conformed to HWE in the control group.

∗ *P-*value less than .05 was considered as statistically significant; ESCC, esophageal squamous cell carcinoma; EAC, esophageal adenocarcinoma.

#### 3.2.2 Association between MMP2 rs243865 polymorphism and rs2285053 polymorphism and ESCC susceptibility

**Figure 3:**
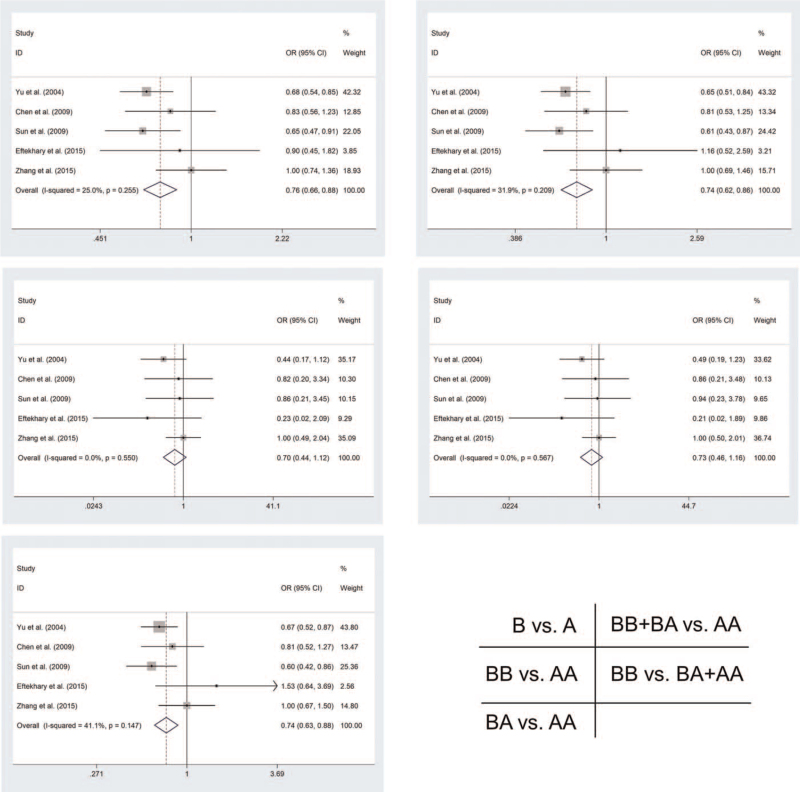
Forest plot showing the risk of MMP2-rs243865 polymorphism to EC risk.

As for rs243865 polymorphism, the study strongly showed that rs243865 polymorphism reduces the risk of ESCC in three models among the overall analysis [B vs. A (OR = 0.761, 95%Cl = 0.659–0.88, *P* < .001); BA vs. AA (OR = 0.743, 95%Cl = 0.628–0.879, *P* = .001); BB + BA vs. AA (OR = 0.735, 95%Cl = 0.625–0.865, *P* < .001)]. We got the same results as the overall analysis in the subgroup analysis of PB [B vs. A (OR = 0.706, 95%Cl = 0.598–0.832, *P* < .001); BA vs. AA (OR = 0.698, 95%Cl = 0.58–0.841, *P* < .001); BB + BA vs. AA (OR = 0.686, 95%Cl = 0.572–0.822, *P* < .001)] and Y [B vs. A (OR = 0.756, 95%Cl = 0.652–0.876, *P* < .001); BA vs. AA (OR = 0.722, 95%Cl = 0.608–0.857, *P* < .001); BB + BA vs. AA (OR = 0.721, 95%Cl = 0.61–0.851, *P* < .001)] (Table 2 and Fig. 3).

In regard to rs2285053 polymorphism, the risk of ESCC and rs2285053 polymorphism is irrelevant (B vs. A [OR = 1.015, 95%Cl = 0.804–1.28, *P* = 0.903]; BB vs. AA [OR = 1.13, 95% Cl = 0.658–1.938, *P* = 0.658]; BA vs. AA [OR = 0.971, 95%Cl = 0.764–1.235, *P* = 0.812]; BB + BA vs. AA [OR = 0.993, 95% Cl = 0.764–1.292, *P* = 0.961]; BB vs. BA + AA [OR = 1.074, 95%Cl = 0.781–1.477, *P* = 0.659]).

#### 3.2.3 Association between MMP3 rs3025058 polymorphism and EC susceptibility

According to our calculations, rs3025058 polymorphism was not associated with the risk of EC in the overall analysis. (B vs. A [OR = 0.926, 95% Cl = 0.698–1.227, *P* = 0.592]; BB vs. AA [OR = 0.875, 95% Cl = 0.426–1.798, *P* = 0.716]; BA vs. AA [OR = 0.959, 95% Cl = 0.583–1.578, *P* = .87]; BB + BA vs. AA [OR = 0.923, 95% Cl = 0.537–1.584, *P* = .771]; BB vs. BA + AA [OR = 0.89, 95% Cl = 0.641–1.234, *P* = .484]). Additionally, we did not find any important association between rs3025058 polymorphism and EC in the subgroup analysis of Asians, Caucasian, ESCC, EAC, Y, and N.

#### 3.2.4 Association between MMP7 rs11568818 polymorphism and ESCC susceptibility

As for rs11568818 polymorphism, the study strongly showed that rs11568818 polymorphism can increase the risk of ESCC among the overall analysis (B vs. A [OR = 1.578, 95%Cl = 1.219–2.044, *P* = .001]; BB vs. AA [OR = 2.068, 95% Cl = 1.166–3.669, *P* = .013]; BB + BA vs. AA [OR = 1.538, 95% Cl = 1.091–2.168, *P* = .014]; BB vs. BA + AA [OR = 2.108, 95%Cl = 1.295–3.431, *P* = .003]).

#### 3.2.5 Association between MMP9 rs3918242 polymorphism, rs2250889 polymorphism, and rs17576 polymorphism and ESCC susceptibility

Unfortunately, we only found evidence that it reduced the risk of ESCC in one model of the overall analysis of the rs2250889 polymorphism (BA vs. AA [OR = 0.354, 95%Cl = 0.215–0.582, *P* < .001]), and the results of this meta-analysis of rs2250889 polymorphism and rs17576 polymorphism showed that these two genetic polymorphisms do not have a relationship with the risk of ESCC (Table 2).

#### 3.2.6 Association between MMP12 rs2276109 polymorphism and rs652438 polymorphism and EC susceptibility

As for rs2276109 polymorphism, the result of subgroup analysis of Caucasian showed that rs2276109 polymorphism increased the risk of EAC (B vs. A [OR = 1.314, 95%Cl = 1.031–1.675, *P* = .027]; BB + BA vs. AA [OR = 1.333, 95% Cl = 1.022–1.738, *P* = .034]).

However, the analysis results for rs652438 polymorphism and the risk of EAC are negative (B vs. A [OR = 1.692, 95%Cl = 0.706–4.053, *P* = .238]; BB vs. AA [OR = 3.171, 95% Cl = 0.331–30.386, *P* = .317]; BA vs. AA [OR = 1.169, 95%Cl = 0.827–1.652, *P* = .376]; BB + BA vs. AA [OR = 1.506, 95% Cl = 0.833–2.724, *P* = .175]; BB vs. BA + AA [OR = 3.121, 95%Cl = 0.329–29.584, *P* = .321]).

#### 3.2.7 Association between MMP13 rs2252070 polymorphism ESCC susceptibility

In regard to rs2252070 polymorphism, the overall *P* values and odds ratios demonstrate no significant rising or declining link between MMP13 rs2252070 polymorphism and cancer risk, whatever the genotype (B vs. A [OR = 1.009, 95%Cl = 0.882–1.154, *P* = .894]; BB vs. AA [OR = 1.021, 95% Cl = 0.777–1.343, *P* = .88]; BA vs. AA [OR = 0.986, 95%Cl = 0.785–1.238, *P* = .902]; BB + BA vs. AA [OR = 0.996, 95% Cl = 0.803–1.236, *P* = .974]; BB vs. BA + AA [OR = 1.031, 95%Cl = 0.821–1.295, *P* = .793]).

### 3.3 Sensitivity analysis and publication bias

**Figure 4:**
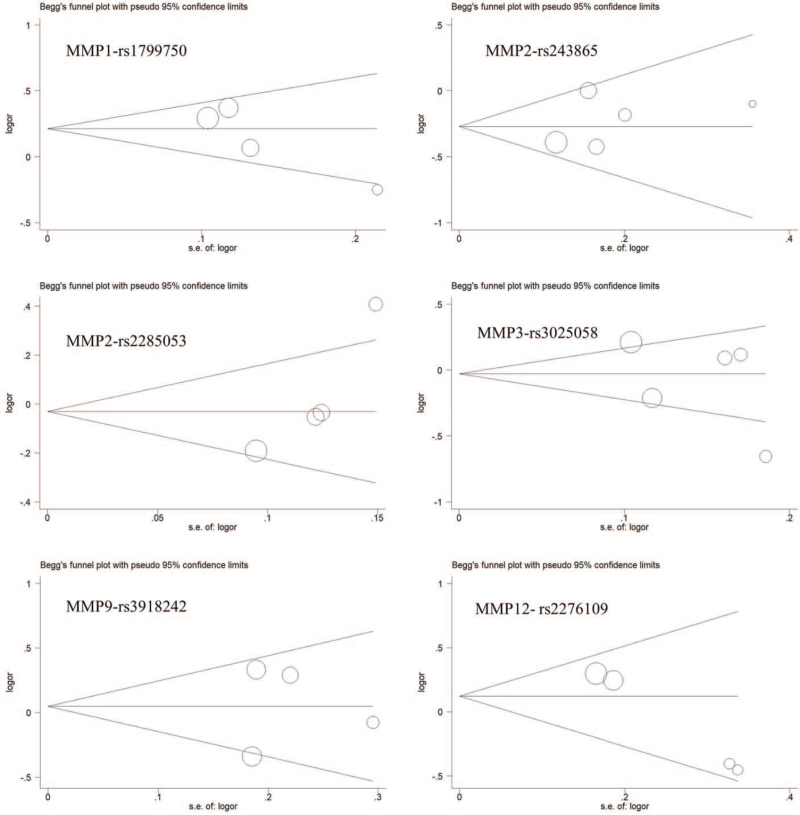
Begg's funnel plot for publication bias for MMPs polymorphisms.

To test the robustness of the results we obtained, we delete individual studies one by one when conducting sensitivity analysis. We have not observed any significant changes in OR and the corresponding 95% CI, which supported that the results of the current study are stable and representative (see Supplementary Digital Content Table S2, http://links.lww.com/MD2/A463 which illustrates the details of sensitivity analysis). What's more, Begg test was used to evaluate the publication bias for the selected literature, the symmetric channel distribution plan shows that there is no prejudice in the publication (Fig. 4). Egger test was also used to analyze the published bias further and shows that no clear evidence of publication bias was found in our study (see Supplementary Digital Content Table S3, http://links.lww.com/MD2/A464 which illustrates the results of Egger test).

### 3.4 MMPs mRNA levels increased in tumor tissues and affected by polymorphisms

**Figure 5:**
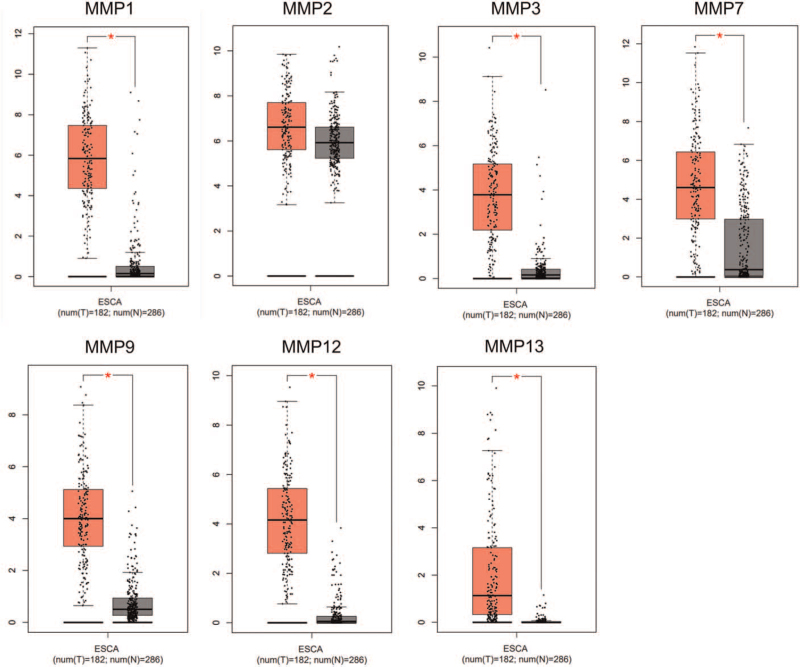
The mRNA expression of MMPs among normal and tumors tissues in EC patients.

Based on the above results, we already knew that the polymorphism of some MMPs could alter the risk score of EC. Based on the TCGA and GTEx data, we found that MMP1, MMP3, MMP7, MMP9, MMP12, MMP13 all increased in the tumor as compared with the normal esophageal tissues (Fig. 5).

## 4 Discussion

The zinc-dependent MMPs are endopeptidases known for their ability to cleave the conditions of the extracellular matrix, of which could help the tumor cells to penetrate and infiltrate into the subjacent stromal matrix, and finally lead to tumor invasion and metastasis.^[30,31]^ There are 28 members in the MMP family, and 23 of them are expressed in human tissues, typically MMPs share common structures, including a ∼80 amino acid propeptide, a ∼170 amino acid catalytic metalloproteinase domain, a hinge region with variable size, and a ∼200 amino acids hemopexin domain.^[32,33]^ Based on the different structures of MMPs, they share different functions. MMP1, MMP8, and MMP13 are human collagenases, MMP3 and MMP10 are tromelysins, plus MMP12, 19, 20, and 27 are all archetypal MMPs; MMP7 and 26 are matrilysins because of the lack of hemopexin domain; MMP2 and MMP9 incorporate three fibronectin type II modules that provide a compact collagen binding domain as the gelatinases; And the three secreted MMP11, 21 and 28 proteins and two unusual transmembrane MMP23A and MMP23B all share the Furin-like cleavage site.^[34]^ In the past decades, increasing evidence confirmed the functions of MMPs in pathological processes, including inflammation, vascular disease, emphysema, liver fibrosis, as well as tumors.^[31,35–38]^

What's more, the polymorphisms of MMPs were also concerned to predict the risk of tumorigenesis in several studies. Białkowska et al^[39]^ reported that the polymorphism rs11568818 in MMP7 gene was also associated with a 2.39-fold increased prostate cancer risk in Poland, while Meng et al^[40]^ also, compared the different effects of MMP polymorphisms in urinary cancer, they revealed that the rs3918241, rs2250889, rs17576, and rs17577 polymorphisms of MMP9 are not associated with altered risk of urinary cancer. As to MMP8, Shen et al^[41]^ reported that the rs11225395, rs34009635, and rs35866072 polymorphisms are not associated with the increased risk of lung cancer in Taiwan district, however, González-Arriaga et al^[42]^ found that the rs215502 of MMP8 acted as a protective role and decreased the lung cancer risk in Spanish. In breast cancer, Wang et al^[43]^ reporting the increased frequency of MMP8 rs11225394 polymorphism, while the decreased frequency of MMP8 rs3787268 polymorphism, these results indicated the internal conflict of MMP8 gene with the risk of breast cancer. Numerous studies also evaluated the predicted value of MMPs polymorphisms in EC risk, however the results are not consistent, therefore, we designed the current study to remove the cloud.

In the current study, we performed a comprehensive search to enroll all the available data about the polymorphisms of MMPs and EC risk. Finally, 19 case-control researches, including 8371 EC patients and 12041 health controls were recorded for the subsequent analysis. The odds ratios and 95% confidence intervals in five genetic models were conducted to evaluate the risk of EC. We observed the increased risk in the heterozygote comparison model and the dominant comparison model in MMP1-rs1799750 polymorphism, as well as in the EAC/Caucasian subgroups. The shielding effectiveness of EC was found in the MMP2 rs243865 polymorphism in B vs. A, BA vs. AA, and BB + BA vs. AA models. Meanwhile, the risk effect was also observed in the MMP7 rs11568818 polymorphism in most genetic models. And the polymorphisms in other MMPs showed no function in the tumorigenesis of EC. In the furthermore bioinformatics analysis, we found that MMP1, MMP3, MMP7, MMP9, MMP12, MMP13 all increased in the tumor as compared with the normal esophageal tissues. In a prior study, Przybylowska et al^[44]^ reported that the altered allele 2G/2G of MMP-1 rs1799750 leads the significantly higher levels of MMP-1 expression than the major 1G/1G, and we also observed the higher level of MMP-1 in EC than normal tissues. For the MMP-7 rs1156881, Gerger et al^[45]^ also reported that the altered G allele can lead to a higher promoter activity. Therefore, we can derive the correlation of polymorphism, MMPs expression and tumorigenesis.

The current study concerned all the applicable studies to analyze the association between MMPs polymorphisms and EC, and showing some advantages. First, we enrolled all eligible studies based on the language of English and Chinese, the race of Asian and Caucasian to conduct a comprehensive study to obtain stable results. Second, the NOS method is used to evaluate the quality of each case-control study. At the same time, we eliminated low-quality studies to ensure the reliability of the combined results. Third,

The stability of the results was evaluated by sensitivity analysis, and the results of Egger test and Begg's funnel chart confirmed that the research results were not biased. On the contrary, there are several disadvantages for the current study. First of all, due to the lack of environmental factors that may affect the phenotype, unreliable results may be obtained. What's more, in about half of the concerned polymorphisms, there are only two or three eligible studies enrolled, therefore, the results of these polymorphisms might be inconvincible. Finally, although we conducted the meta-analysis using the Der Simonian and Laird methods, the heterogeneity between recorded publications may affect the results.

## 5 Conclusion

All in all, based on the results in the current study, we can draw out that MMP1 rs1799705 and MMP7 rs1156818 polymorphisms will take part in the tumorigenesis of EC, while MMP2 rs243865 acts as a protective role to decrease the risk of EC. More studies are needed to confirm the predicted value of MMPs polymorphisms to the risk of EC.

## Author contributions

Conceived and designed the study: Hai Chen; Performed the literature search and data extraction: Hai Chen, Zaicheng Yu, Congshu Hua, Tengfei Ge; Analyzed the data: Heng Zhang, Junling Jian, Jianfeng Xie; Drafted the manuscript: Hai Chen, Xianquan Xu.

**Conceptualization:** Hai Chen.

**Data curation:** Congshu Hua, Heng Zhang, Junling Jian, Tengfei Ge, Jianfeng Xie.

**Formal analysis:** Junling Jian, Jianfeng Xie.

**Investigation:** Congshu Hua, Heng Zhang, Tengfei Ge.

**Project administration:** Zaicheng Yu.

**Resources:** Congshu Hua, Heng Zhang, Tengfei Ge.

**Writing – original draft:** Hai Chen, Xianquan Xu.

**Writing – review & editing:** Zaicheng Yu.

## Supplementary Material

SUPPLEMENTARY MATERIAL
