# Supplementary material for: Polymorphisms of matrix metalloproteinases affect the susceptibility of esophageal cancer: Evidence from 20412 subjects, systematic review and updated meta-analysis
Source: Medicine (Baltimore). 2021 Sep 24;100(38):e27229. doi: 10.1097/MD.0000000000027229 (PMC10545374; doi:10.1097/MD.0000000000027229)
Supplement: SUPPLEMENTARY MATERIAL [file medi-100-e27229-s001.doc]

**Table S1. Methodological quality of the included studies according to the Newcastle-Ottawa Scale.**

| First author | Year | Adequacy of Case Definition | Representative-  ness of the Cases | Selection of Controls | Definition of Controls | Comparability Cases/Controls | Ascertainment of Exposure | Same Method of Ascertainment | Non-response rate |
| --- | --- | --- | --- | --- | --- | --- | --- | --- | --- |
|
| Zhang et al. | 2007 | * | * | * | * | * | * | * | NA |
| Sun et al. | 2009 | * | * | * | * | ** | * | * | NA |
| Gao et al. | 2011 | * | * | * | * | * | * | * | NA |
| Liu et al. | 2012 | * |  | * | * |  | * | * | NA |
| Fu et al. | 2009 | * | * | * | * | ** | * | * | NA |
| Ouyang et al. | 2009 | * | * | * | * |  | * | * | NA |
| Bradbury et al. | 2009 | * | * | * | * | * | * | * | NA |
| Chen et al. | 2009 | * | * | * | * | * | * | * | NA |
| Cheung et al. | 2012 | * | * | * | * | ** | * | * | NA |
| Eftekhary et al. | 2015 | * | * | * | * |  | * | * | NA |
| Guan et al. | 2014 | * | * | * | * | ** | * | * | NA |
| Li et al. | 2010 | * | * | * | * | * | * | * | NA |
| Malik et al. | 2011 | * | * | * | * | ** | * | * | NA |
| Wu et al. | 2008 | * | * | * | * | ** | * | * | NA |
| Jin et al. | 2005 | * | * |  |  | * | * | * | NA |
| Yu et al. | 2004 | * | * | * | * | * | * | * | NA |
| Zhang et al. | 2004 | * | * | * | * | * | * | * | NA |
| Zhang et al. | 2005 | * | * | * | * |  | * | * | NA |
| Zhang et al. | 2015 | * | * |  |  | * | * | * | NA |

This table identifies “high” quality choices with a “star”. A study can be awarded a maximum of 1 star for each numbered item within the Selection and Exposure categories. A maximum of 2 stars can be given for Comparability. *, Yes; NA, not applicable. (http://www.ohri.ca/programs/clinical epidemiology/oxford.htm).
