# Supplementary material for: Polymorphisms of matrix metalloproteinases affect the susceptibility of esophageal cancer: Evidence from 20412 subjects, systematic review and updated meta-analysis
Source: Medicine (Baltimore). 2021 Sep 24;100(38):e27229. doi: 10.1097/MD.0000000000027229 (PMC10545374; doi:10.1097/MD.0000000000027229)
Supplement: SUPPLEMENTARY MATERIAL [file medi-100-e27229-s002.doc]

**Table S2. Details of the sensitivity analyses for MMPs polymorphism and cancer risk.**

| **Polymorphism** | **Comparison** | **Study omitted** | **Estimate (95% Confident Interval)** | **Effect Model** |
| --- | --- | --- | --- | --- |
| MMP1-rs1799750 | B vs. A | Jin et al. (2005) | 1.212( 0.913-1.61) | Random |
|  |  | Bradbury et al. (2009) | 1.103( 0.798-1.526) |  |
|  |  | Cheung et al. (2012) | 1.087( 0.825-1.43) |  |
|  |  | Guan et al. (2014) | 1.289( 1.092-1.521) |  |
|  |  | Combined | 1.182( 0.95-1.471) |  |
|  | BB vs. AA | Jin et al. (2005) | 1.477( 0.834-2.616) | Random |
|  |  | Bradbury et al. (2009) | 1.236( 0.634-2.409) |  |
|  |  | Cheung et al. (2012) | 1.207( 0.694-2.101) |  |
|  |  | Guan et al. (2014) | 1.672( 1.261-2.218) |  |
|  |  | Combined | 1.44( 0.944-2.195) |  |
|  | BA vs. AA | Jin et al. (2005) | 1.327( 1.035-1.7) | Fixed |
|  |  | Bradbury et al. (2009) | 1.257( 0.931-1.696) |  |
|  |  | Cheung et al. (2012) | 1.305( 0.986-1.728) |  |
|  |  | Guan et al. (2014) | 1.38( 1.095-1.739) |  |
|  |  | Combined | 1.325( 1.057-1.661) |  |
|  | BB+BA vs. AA | Jin et al. (2005) | 1.449( 1.148-1.827) | Fixed |
|  |  | Bradbury et al. (2009) | 1.336( 1.012-1.765) |  |
|  |  | Cheung et al. (2012) | 1.341( 1.034-1.739) |  |
|  |  | Guan et al. (2014) | 1.476( 1.19-1.831) |  |
|  |  | Combined | 1.411( 1.143-1.741) |  |
|  | BB vs. BA+ AA | Jin et al. (2005) | 1.291( 0.885-1.885) | Random |
|  |  | Bradbury et al. (2009) | 1.137( 0.753-1.718) |  |
|  |  | Cheung et al. (2012) | 1.086( 0.812-1.452) |  |
|  |  | Guan et al. (2014) | 1.321( 0.978-1.785) |  |
|  |  | Combined | 1.207( 0.898-1.623) |  |
| MMP12-rs2276109 | B vs. A | Zhang et al. (2007) | 1.15( 0.824-1.605) | Random |
|  |  | Bradbury et al. (2009) | 0.868( 0.525-1.433) |  |
|  |  | Li et al. (2010) | 1.137( 0.801-1.616) |  |
|  |  | Cheung et al. (2012) | 0.882( 0.508-1.532) |  |
|  |  | Combined | 1.018( 0.712-1.455) |  |
|  | BA vs. AA | Zhang et al. (2007) | 1.126( 0.783-1.618) | Random |
|  |  | Bradbury et al. (2009) | 0.851( 0.529-1.37) |  |
|  |  | Li et al. (2010) | 1.113( 0.759-1.631) |  |
|  |  | Cheung et al. (2012) | 0.877( 0.498-1.547) |  |
|  |  | Combined | 0.996( 0.684-1.451) |  |
|  | BB+BA vs. AA | Zhang et al. (2007) | 1.143( 0.794-1.646) | Random |
|  |  | Bradbury et al. (2009) | 0.858( 0.52-1.417) |  |
|  |  | Li et al. (2010) | 1.13( 0.77-1.658) |  |
|  |  | Cheung et al. (2012) | 0.88( 0.495-1.564) |  |
|  |  | Combined | 1.008( 0.688-1.475) |  |
| MMP12-rs652438 | B vs. A | Bradbury et al. (2009) | 1.067( 0.665-1.71) | Random |
|  |  | Cheung et al. (2012) | 2.602( 1.846-3.669) |  |
|  |  | Combined | 1.692( 0.706-4.053) |  |
|  | BB vs. AA | Bradbury et al. (2009) | 0.912( 0.182-4.559) | Random |
|  |  | Cheung et al. (2012) | 9.142( 3.511-23.802) |  |
|  |  | Combined | 3.171( 0.331-30.386) |  |
|  | BA vs. AA | Bradbury et al. (2009) | 1.107( 0.652-1.879) | Fixed |
|  |  | Cheung et al. (2012) | 1.218( 0.772-1.922) |  |
|  |  | Combined | 1.169( 0.827-1.652) |  |
|  | BB+BA vs. AA | Bradbury et al. (2009) | 1.088( 0.655-1.807) | Random |
|  |  | Cheung et al. (2012) | 1.995( 1.346-2.955) |  |
|  |  | Combined | 1.506( 0.833-2.724) |  |
|  | BB vs. BA+ AA | Bradbury et al. (2009)) | 0.902( 0.181-4.506) | Random |
|  |  | Cheung et al. (2012)) | 8.942( 3.439-23.251) |  |
|  |  | Combined | 3.121( 0.329-29.584) |  |
| MMP13-rs2252070 | B vs. A | Zhang et al. (2007) | 1.006( 0.833-1.214) | Fixed |
|  |  | Li et al. (2010) | 1.013( 0.836-1.228) |  |
|  |  | Combined | 1.009( 0.882-1.154) |  |
|  | BB vs. AA | Zhang et al. (2007) | 1.016( 0.694-1.487) | Fixed |
|  |  | Li et al. (2010) | 1.027( 0.693-1.522) |  |
|  |  | Combined | 1.021( 0.777-1.343) |  |
|  | BA vs. AA | Zhang et al. (2007) | 0.961( 0.699-1.321) | Fixed |
|  |  | Li et al. (2010) | 1.013( 0.731-1.403) |  |
|  |  | Combined | 0.986( 0.785-1.238) |  |
|  | BB+BA vs. AA | Zhang et al. (2007) | 0.977( 0.723-1.321) | Fixed |
|  |  | Li et al. (2010) | 1.017( 0.746-1.386) |  |
|  |  | Combined | 0.996( 0.803-1.236) |  |
|  | BB vs. BA+ AA | Zhang et al. (2007) | 1.043( 0.759-1.434) | Fixed |
|  |  | Li et al. (2010) | 1.018( 0.734-1.413) |  |
|  |  | Combined | 1.031( 0.821-1.295) |  |
| MMP2-rs243865 | B vs. A | Yu et al. (2004) | 0.824( 0.684-0.992) | Fixed |
|  |  | Chen et al. (2009) | 0.751( 0.643-0.877) |  |
|  |  | Sun et al. (2009) | 0.792( 0.674-0.931) |  |
|  |  | Eftekhary et al. (2015) | 0.756( 0.652-0.876) |  |
|  |  | Zhang et al. (2015) | 0.706( 0.598-0.832) |  |
|  |  | Combined | 0.761( 0.659-0.88) |  |
|  | BB vs. AA | Yu et al. (2004) | 0.838( 0.483-1.455) | Fixed |
|  |  | Chen et al. (2009) | 0.684( 0.416-1.125) |  |
|  |  | Sun et al. (2009) | 0.68( 0.413-1.119) |  |
|  |  | Eftekhary et al. (2015) | 0.746( 0.46-1.21) |  |
|  |  | Zhang et al. (2015) | 0.535( 0.281-1.016) |  |
|  |  | Combined | 0.698( 0.437-1.116) |  |
|  | BA vs. AA | Yu et al. (2004) | 0.799( 0.641-0.995) | Fixed |
|  |  | Chen et al. (2009) | 0.732( 0.611-0.878) |  |
|  |  | Sun et al. (2009) | 0.791( 0.654-0.957) |  |
|  |  | Eftekhary et al. (2015) | 0.722( 0.608-0.857) |  |
|  |  | Zhang et al. (2015) | 0.698( 0.58-0.841) |  |
|  |  | Combined | 0.743( 0.628-0.879) |  |
|  | BB+BA vs. AA | Yu et al. (2004) | 0.797( 0.645-0.985) | Fixed |
|  |  | Chen et al. (2009) | 0.723( 0.607-0.862) |  |
|  |  | Sun et al. (2009) | 0.775( 0.645-0.932) |  |
|  |  | Eftekhary et al. (2015) | 0.721( 0.61-0.851) |  |
|  |  | Zhang et al. (2015) | 0.686( 0.572-0.822) |  |
|  |  | Combined | 0.735( 0.625-0.865) |  |
|  | BB vs. BA+ AA | Yu et al. (2004) | 0.852( 0.495-1.467) | Fixed |
|  |  | Chen et al. (2009) | 0.714( 0.436-1.169) |  |
|  |  | Sun et al. (2009) | 0.706( 0.431-1.157) |  |
|  |  | Eftekhary et al. (2015) | 0.786( 0.487-1.269) |  |
|  |  | Zhang et al. (2015) | 0.571( 0.301-1.084) |  |
|  |  | Combined | 0.729( 0.458-1.16) |  |
| MMP2-rs2285053 | B vs. A | Yu et al. (2004) | 1.099( 0.835-1.448) | Random |
|  |  | Zhang et al. (2007) | 1.039( 0.749-1.442) |  |
|  |  | Chen et al. (2009) | 0.894( 0.789-1.014) |  |
|  |  | Sun et al. (2009) | 1.046( 0.752-1.454) |  |
|  |  | Combined | 1.015( 0.804-1.28) |  |
|  | BB vs. AA | Yu et al. (2004) | 1.304( 0.599-2.837) | Random |
|  |  | Zhang et al. (2007) | 1.23( 0.574-2.637) |  |
|  |  | Chen et al. (2009) | 0.865( 0.607-1.232) |  |
|  |  | Sun et al. (2009) | 1.287( 0.614-2.7) |  |
|  |  | Combined | 1.13( 0.658-1.938) |  |
|  | BA vs. AA | Yu et al. (2004) | 1.069( 0.86-1.33) | Random |
|  |  | Zhang et al. (2007) | 0.987( 0.703-1.385) |  |
|  |  | Chen et al. (2009) | 0.873( 0.731-1.043) |  |
|  |  | Sun et al. (2009) | 0.979( 0.697-1.375) |  |
|  |  | Combined | 0.971( 0.764-1.235) |  |
|  | BB+BA vs. AA | Yu et al. (2004) | 1.098( 0.832-1.449) | Random |
|  |  | Zhang et al. (2007) | 1.016( 0.7-1.476) |  |
|  |  | Chen et al. (2009) | 0.87( 0.742-1.02) |  |
|  |  | Sun et al. (2009) | 1.016( 0.697-1.48) |  |
|  |  | Combined | 0.993( 0.764-1.292) |  |
|  | BB vs. BA+ AA | Yu et al. (2004) | 1.167( 0.779-1.749) | Fixed |
|  |  | Zhang et al. (2007) | 1.109( 0.773-1.589) |  |
|  |  | Chen et al. (2009) | 0.914( 0.644-1.296) |  |
|  |  | Sun et al. (2009) | 1.164( 0.81-1.674) |  |
|  |  | Combined | 1.074( 0.781-1.477) |  |
| MMP3-rs3025058 | B vs. A | Zhang et al. (2004) | 0.886( 0.625-1.257) | Random |
|  |  | Bradbury et al. (2009) | 0.852( 0.624-1.163) |  |
|  |  | Cheung et al. (2012) | 0.958( 0.672-1.365) |  |
|  |  | Guan et al. (2014) | 1.048( 0.849-1.294) |  |
|  |  | Zhang et al. (2015) | 0.883( 0.627-1.244) |  |
|  |  | Combined | 0.926( 0.698-1.227) |  |
|  | BB vs. AA | Zhang et al. (2004) | 0.745( 0.358-1.549) | Random |
|  |  | Bradbury et al. (2009) | 0.723( 0.301-1.736) |  |
|  |  | Cheung et al. (2012) | 0.988( 0.383-2.547) |  |
|  |  | Guan et al. (2014) | 1.171( 0.648-2.113) |  |
|  |  | Zhang et al. (2015) | 0.819( 0.309-2.167) |  |
|  |  | Combined | 0.875( 0.426-1.798) |  |
|  | BA vs. AA | Zhang et al. (2004) | 0.885( 0.547-1.432) | Random |
|  |  | Bradbury et al. (2009) | 0.819( 0.456-1.468) |  |
|  |  | Cheung et al. (2012) | 1.074( 0.576-2.002) |  |
|  |  | Guan et al. (2014) | 1.117( 0.7-1.783) |  |
|  |  | Zhang et al. (2015) | 0.928( 0.465-1.85) |  |
|  |  | Combined | 0.959( 0.583-1.578) |  |
|  | BB+BA vs. AA | Zhang et al. (2004) | 0.837( 0.49-1.432) | Random |
|  |  | Bradbury et al. (2009) | 0.778( 0.404-1.498) |  |
|  |  | Cheung et al. (2012) | 1.025( 0.52-2.02) |  |
|  |  | Guan et al. (2014) | 1.128( 0.701-1.813) |  |
|  |  | Zhang et al. (2015) | 0.874( 0.402-1.897) |  |
|  |  | Combined | 0.923( 0.537-1.584) |  |
|  | BB vs. BA+ AA | Zhang et al. (2004) | 0.848( 0.548-1.313) | Random |
|  |  | Bradbury et al. (2009) | 0.808( 0.554-1.177) |  |
|  |  | Cheung et al. (2012) | 0.913( 0.601-1.385) |  |
|  |  | Guan et al. (2014) | 1.039( 0.854-1.263) |  |
|  |  | Zhang et al. (2015) | 0.846( 0.577-1.241) |  |
|  |  | Combined | 0.89( 0.641-1.234) |  |
| MMP7-rs11568818 | B vs. A | Zhang et al. (2005) | 1.515( 1.109-2.069) | Fixed |
|  |  | Malik et al. (2011) | 1.727( 1.089-2.74) |  |
|  |  | Combined | 1.578( 1.219-2.044) |  |
|  | BB vs. AA | Zhang et al. (2005) | 2.1( 1.168-3.774) | Fixed |
|  |  | Malik et al. (2011) | 1.463( 0.091-23.516) |  |
|  |  | Combined | 2.068( 1.166-3.669) |  |
|  | BA vs. AA | Zhang et al. (2005) | 0.97( 0.569-1.654) | Random |
|  |  | Malik et al. (2011) | 1.818( 1.114-2.967) |  |
|  |  | Combined | 1.34( 0.725-2.479) |  |
|  | BB+BA vs. AA | Zhang et al. (2005) | 1.313( 0.808-2.133) | Fixed |
|  |  | Malik et al. (2011) | 1.807( 1.114-2.933) |  |
|  |  | Combined | 1.538( 1.091-2.168) |  |
|  | BB vs. BA+ AA | Zhang et al. (2005) | 2.138( 1.303-3.507) | Fixed |
|  |  | Malik et al. (2011) | 1.358( 0.085-21.812) |  |
|  |  | Combined | 2.108( 1.295-3.431) |  |
| MMP9-rs3918242 | B vs. A | Fu et al. (2009) | 0.948( 0.635-1.416) | Random |
|  |  | Guan et al. (2014) | 0.975( 0.626-1.519) |  |
|  |  | Zhang et al. (2015) | 1.27( 0.986-1.635) |  |
|  |  | Eftekhary et al. (2015) | 1.09( 0.701-1.695) |  |
|  |  | Combined | 1.055( 0.746-1.494) |  |
|  | BB vs. AA | Fu et al. (2009) | 0.803( 0.273-2.355) | Random |
|  |  | Guan et al. (2014) | 0.911( 0.218-3.806) |  |
|  |  | Zhang et al. (2015) | 1.766( 0.774-4.033) |  |
|  |  | Eftekhary et al. (2015) | 1.243( 0.287-5.378) |  |
|  |  | Combined | 1.091( 0.364-3.27) |  |
|  | BA vs. AA | Fu et al. (2009) | 0.904( 0.611-1.339) | Fixed |
|  |  | Guan et al. (2014) | 1.026( 0.737-1.43) |  |
|  |  | Zhang et al. (2015) | 1.207( 0.894-1.629) |  |
|  |  | Eftekhary et al. (2015) | 1.106( 0.813-1.505) |  |
|  |  | Combined | 1.076( 0.81-1.429) |  |
|  |  |  | 1.076( 0-0) |  |
|  | BB+BA vs. AA | Fu et al. (2009) | 0.825( 0.417-1.632) | Random |
|  |  | Guan et al. (2014) | 0.846( 0.417-1.718) |  |
|  |  | Zhang et al. (2015) | 1.264( 0.946-1.689) |  |
|  |  | Eftekhary et al. (2015) | 0.984( 0.527-1.839) |  |
|  |  | Combined | 0.993( 0.619-1.593) |  |
|  | BB vs. BA+ AA | Fu et al. (2009) | 0.904( 0.619-1.322) | Fixed |
|  |  | Guan et al. (2014) | 0.888( 0.596-1.322) |  |
|  |  | Zhang et al. (2015) | 1.765( 0.797-3.908) |  |
|  |  | Eftekhary et al. (2015) | 0.974( 0.666-1.425) |  |
|  |  | Combined | 0.969( 0.669-1.403) |  |
| MMP9-rs2250889 | B vs. A | Wu et al. (2008) | 1.583( 1.203-2.082) | Random |
|  |  | Fu et al. (2009) | 0.598( 0.417-0.856) |  |
|  |  | Combined | 0.98( 0.377-2.543) |  |
|  | BB vs. AA | Wu et al. (2008) | 1.19( 0.712-1.99) | Random |
|  |  | Fu et al. (2009) | 0.245( 0.095-0.631) |  |
|  |  | Combined | 0.569( 0.121-2.674) |  |
|  | BA vs. AA | Wu et al. (2008) | 0.344( 0.191-0.618) | Fixed |
|  |  | Fu et al. (2009) | 0.377( 0.15-0.95) |  |
|  |  | Combined | 0.354( 0.215-0.582) |  |
|  | BB+BA vs. AA | Wu et al. (2008) | 0.804( 0.489-1.322) | Random |
|  |  | Fu et al. (2009) | 0.314( 0.128-0.77) |  |
|  |  | Combined | 0.542( 0.218-1.349) |  |
|  | BB vs. BA+ AA | Wu et al. (2008) | 2.389( 1.672-3.412) | Random |
|  |  | Fu et al. (2009) | 0.56( 0.339-0.925) |  |
|  |  | Combined | 1.17( 0.282-4.845) |  |
| MMP9-rs17576 | B vs. A | Wu et al. (2008) | 1.282( 1.006-1.635) | Fixed |
|  |  | Fu et al. (2009) | 0.904( 0.622-1.313) |  |
|  |  | Combined | 1.156( 0.943-1.416) |  |
|  | BB vs. AA | Wu et al. (2008) | 1.434( 0.92-2.237) | Fixed |
|  |  | Fu et al. (2009) | 0.646( 0.236-1.768) |  |
|  |  | Combined | 1.255( 0.838-1.878) |  |
|  | BA vs. AA | Wu et al. (2008) | 0.761( 0.492-1.177) | Fixed |
|  |  | Fu et al. (2009) | 0.647( 0.235-1.783) |  |
|  |  | Combined | 0.741( 0.497-1.106) |  |
|  | BB+BA vs. AA | Wu et al. (2008) | 1.025( 0.693-1.514) | Fixed |
|  |  | Fu et al. (2009) | 0.647( 0.243-1.721) |  |
|  |  | Combined | 0.961( 0.669-1.379) |  |
|  | BB vs. BA+ AA | Wu et al. (2008) | 1.691( 1.179-2.424) | Random |
|  |  | Fu et al. (2009) | 0.944( 0.585-1.521) |  |
|  |  | Combined | 1.291( 0.73-2.283) |  |
