# Supplementary material for: Polymorphisms of matrix metalloproteinases affect the susceptibility of esophageal cancer: Evidence from 20412 subjects, systematic review and updated meta-analysis
Source: Medicine (Baltimore). 2021 Sep 24;100(38):e27229. doi: 10.1097/MD.0000000000027229 (PMC10545374; doi:10.1097/MD.0000000000027229)
Supplement: SUPPLEMENTARY MATERIAL [file medi-100-e27229-s003.doc]

**Table S3. P values of the Egger’s test for MMPs polymorphism.**

| Polymorphisms | Subgroup | Egger’s test P > |t| |
| --- | --- | --- |
| MMP1-rs1799750 | Overall | 0.115 |
|  | PB/Y | 0.242 |
| MMP12- rs2276109 | Overall | 0.002 |
| MMP2-rs243865 | Overall | 0.495 |
|  | PB | 0.214 |
|  | Y | 0.576 |
| MMP2-rs2285053 | Overall | 0.079 |
| MMP3-rs3025058 | Overall | 0.427 |
|  | Asians/ESCC | 0.284 |
|  | Y | 0.382 |
| MMP9-rs3918242 | Overall | 0.957 |
|  | PB | 0.149 |

Abbreviations: P-B, population-based; PH, P-value of Q test for heterogeneity test; Y, polymorphisms conformed to HWE in the control group.; ESCC, esophageal squamous cell carcinoma.
